# Supplementary material for: Tuberculosis commodities supply chain performance in the WHO African region: A scoping review
Source: PLOS Glob Public Health. 2024 May 16;4(5):e0003219. doi: 10.1371/journal.pgph.0003219 (PMC11098481; doi:10.1371/journal.pgph.0003219)
Supplement: Attachment — Submitted filename: Response to reviewers.docx [file pgph.0003219.s002.docx]

**Response to reviewers**

**Reviewer #1:**

1. The logistics cycle diagram should be labelled as a figure.

**Response:** This has been done. See page 7. However, although the figure is the first one in the paper, it has not been named as figure 1 since the journal editorial recommended that that the PRISMA flow diagram should be labelled as figure 1

2. Table 1 -in the strategy column the “The political declaration of the UN High Level Meeting on the fight against tuberculosis” is not dated as the other strategies in the table on page 10

**Response:** Addressed, dated 2023

3. The authors mentioned four journal databases were used for the literature search however only three are name on page 12. The fourth database should be added.

**Response: Three databases were searched. That has been aligned.**

4. The footnotes related to asterisk within the PRISMA flow diagram are missing on page 15

**Response:** For easy reference, the asterisks have been removed and the related description provided in the main PRISMA flow diagram

**Reviewer #2:**

1. DOT(S) is a bit obsolete terminology in the TB program, as apart from the direct observation strategy other modes of administration are used (family-, community-, self -administration).

**Response**: This is acknowledged here. However, family and community based DOTS remain variants/improvements in the main DOTS strategy. To align with that, the authors elected to refer to DOTS in the paper, a terminology that continues to permeate the global strategies for TB control. The following statement has been added to provide context:

*“Although the initial framing on DOTS was centered on health worker supervision for TB chemotherapy, more patient centered models of TB care such as family member and community based DOTS have also emerged as effective strategies in TB control. Since its launch, DOTS and its emergent variants have remained a cornerstone for controlling TB globally(5) and it has informed successive frameworks and targets for international and national efforts aimed at controlling the disease”*

2. Some good points mentioned in the part of the "Areas of further research" which needed to be highlighted and be considered in the Limitations.

**Response:** This has been done. The following have been moved from areas of further research to limitations

1) First, the corpus of studies on TB supply chain performance is generally low which constrains effective pooling of knowledge in the area. However, our scoping review found an encouraging trend of PSM publications from countries such as Ethiopia, and other countries could learn from the country’s experience regarding TB supply chain studies.
2) The other limitation is that most reviewed studies are descriptive in nature. This leads to vague generalizations of PSM performance such as poor ‘logistics’ followed by imprecise and non-actionable recommendations for improving supply chain performance.
