# Supplementary material for: Tuberculosis commodities supply chain performance in the WHO African region: A scoping review
Source: PLOS Glob Public Health. 2024 May 16;4(5):e0003219. doi: 10.1371/journal.pgph.0003219 (PMC11098481; doi:10.1371/journal.pgph.0003219)
Supplement: Attachment — Submitted filename: Response to reviewers.docx [file pgph.0003219.s003.docx]

**Response to reviewers**

1.Please review your reference list to ensure that it is complete and correct. If you have cited papers that have been retracted, please include the rationale for doing so in the manuscript text, or remove these references and replace them with relevant current references. Any changes to the reference list should be mentioned in the rebuttal letter that accompanies your revised manuscript. If you need to cite a retracted article, indicate the article’s retracted status in the References list and also include a citation and full reference for the retraction notice.

**Response**. This has been done. 12 references have been updated: reference 1,18,16, 20, 21,34,35,46,47,49,59,60. There is no citation of a retracted article.

2.We have noticed that you have uploaded Supporting Information files, but you have not included a list of legends. Please add a full list of legends for your Supporting Information files after the references list.

**Response**. This has been done. Relevant legend inserted after the references list.
